# Supplementary material for: Complexation Nanoarchitectonics of Carbon Dots with Doxorubicin toward Photodynamic Anti-Cancer Therapy
Source: J Funct Biomater. 2022 Nov 5;13(4):219. doi: 10.3390/jfb13040219 (PMC9680231; doi:10.3390/jfb13040219)
Supplement: Supplementary file 1 [file jfb-13-00219-s001.zip › jfb-1904168-supplementary.pdf]

Supporting Information

Complexation Nanoarchitectonics of Carbon Dots with Doxorubicin toward Photodynamic Anti-Cancer Therapy

Thu Thi Anh Do,[1] Kukuh Wicaksono,[1] Andree Soendoro,[2] Toyoko Imae,[1,2]* María José Garcia-Celma,[3,4] Santiago Grijalvo[4]

[1]Graduate Institute of Applied Science and Technology, National Taiwan University of Science and Technology, 43 Section 4, Keelung Road, Taipei 10607, Taiwan, Republic of China.

[2]Department of Chemical Engineering, National Taiwan University of Science and Technology, 43 Section 4, Keelung Road, Taipei 10607, Taiwan, Republic of China.

[3]Department of Pharmacy and Pharmaceutical Technology and Physicochemistry, Faculty of Pharmacy and Food Sciences. Institute of Nanoscience and Nanotechnology (IN2UB). University of Barcelona, Joan XXIII s/n, 08028, Barcelona,

[4]Networking Research Center on Bioengineering, Biomaterials and Nanomedicine (CIBER-BBN), Jordi Girona 18-26 08034, Barcelona, Spain.

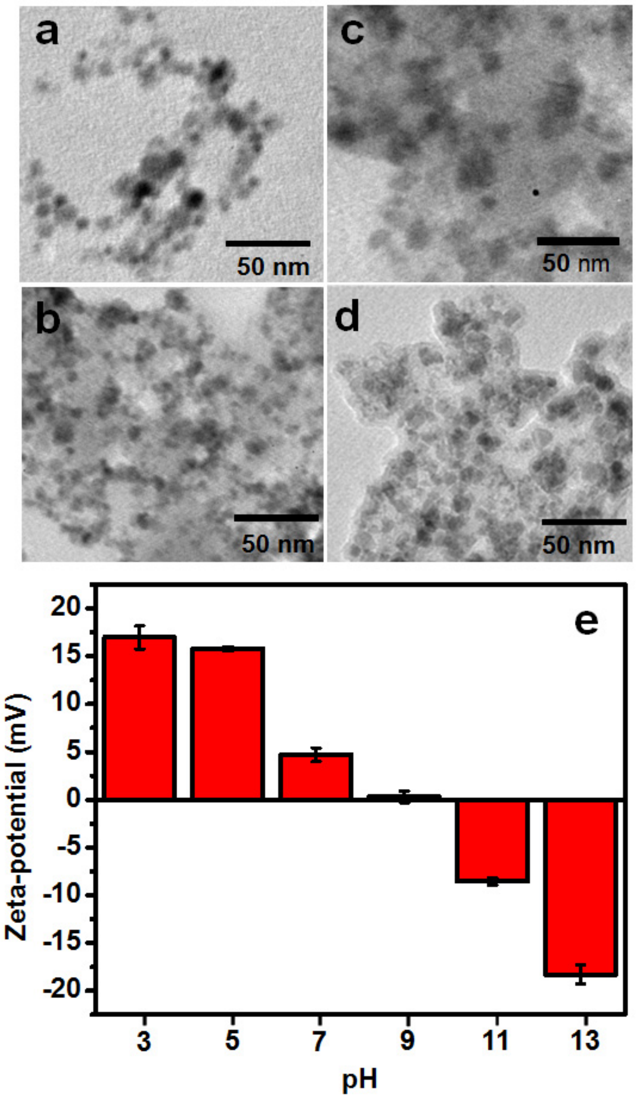

Figure S1. TEM images of (a) Cdots1:1/DOX, (b) Cdots1:2/DOX, (c) Cdots1:3/DOX, and (d) Cdots1:5/DOX and (e) Zeta-potential of DOX.

Anthracycline antibiotics including doxorubicin are known to self-aggregate in water. Then aggregates can present zeta potential. Palai et al. [1] also reported the zeta potential of free DOX.

Reference

1. Palai, P.K., Mondal, A., Chakraborti, C.K., Banerjee, I., Pal, K. and Rathnam, V. Doxorubicin Loaded Green Synthesized Nanoceria Decorated Functionalized Graphene Nanocomposite for Cancer-Specific Drug Release. *J. Clust. Sci.* **2019**, *30*, 1565–1582. https://doi.org/10.1007/s10876-019-01599-4.

Table S1. Binding energies obtained from XPS of Cdots and their assignments.

Element	Cdots1:1	Cdots1:1.5	Assignment
C1s	284.8	284.7	CC(aromatic)
	285.3	285.9	C-OOH/C-NH_2(amine)
	287.5	288.1	CC(alkyl)
	288.6	289.0	-C-OH(hydroxyl)
N1s	399.9	399.7	-C-NH_2(amine)
O1s	532.2	531.9	-C=O(COOH)
	533.1	533.3	-C-OH(COOH)

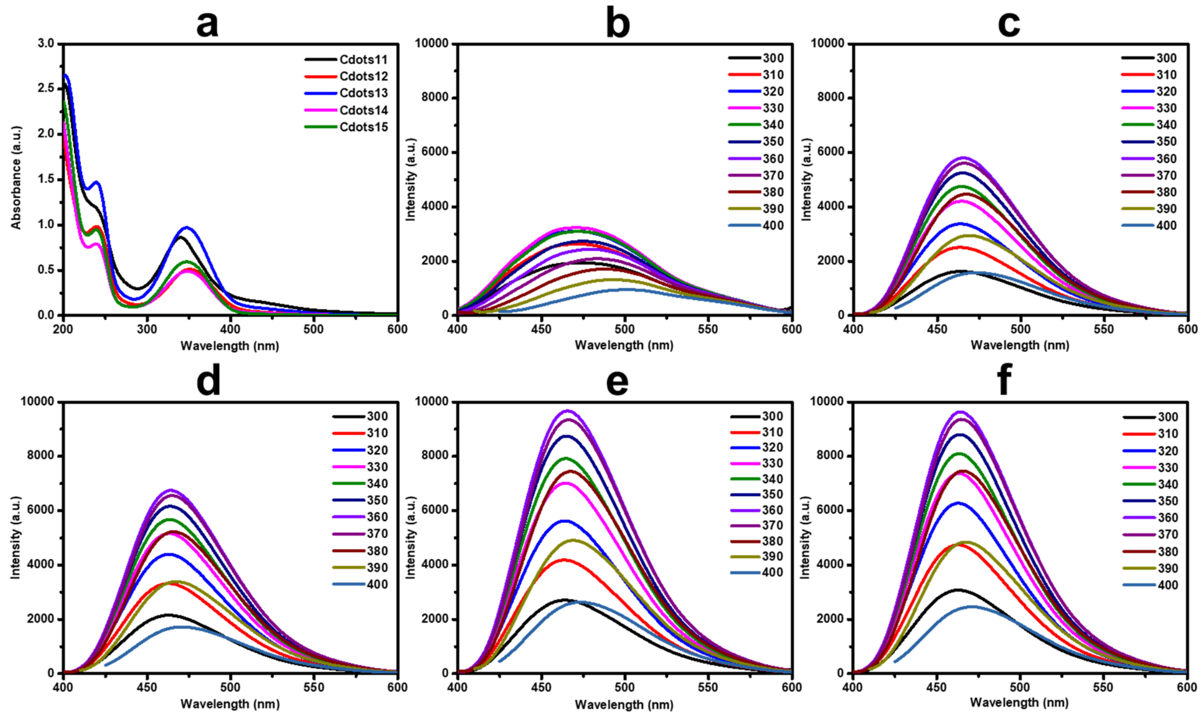

Figure S2. (a) UV-visible absorption spectra of Cdots1:x and emission spectra of (b) Cdots1:1, (c) Cdots1:2, (d) Cdots1:3, (e) Cdots1:4 and (f) Cdots1:5 at different excitation wavelengths (300 - 400 nm).

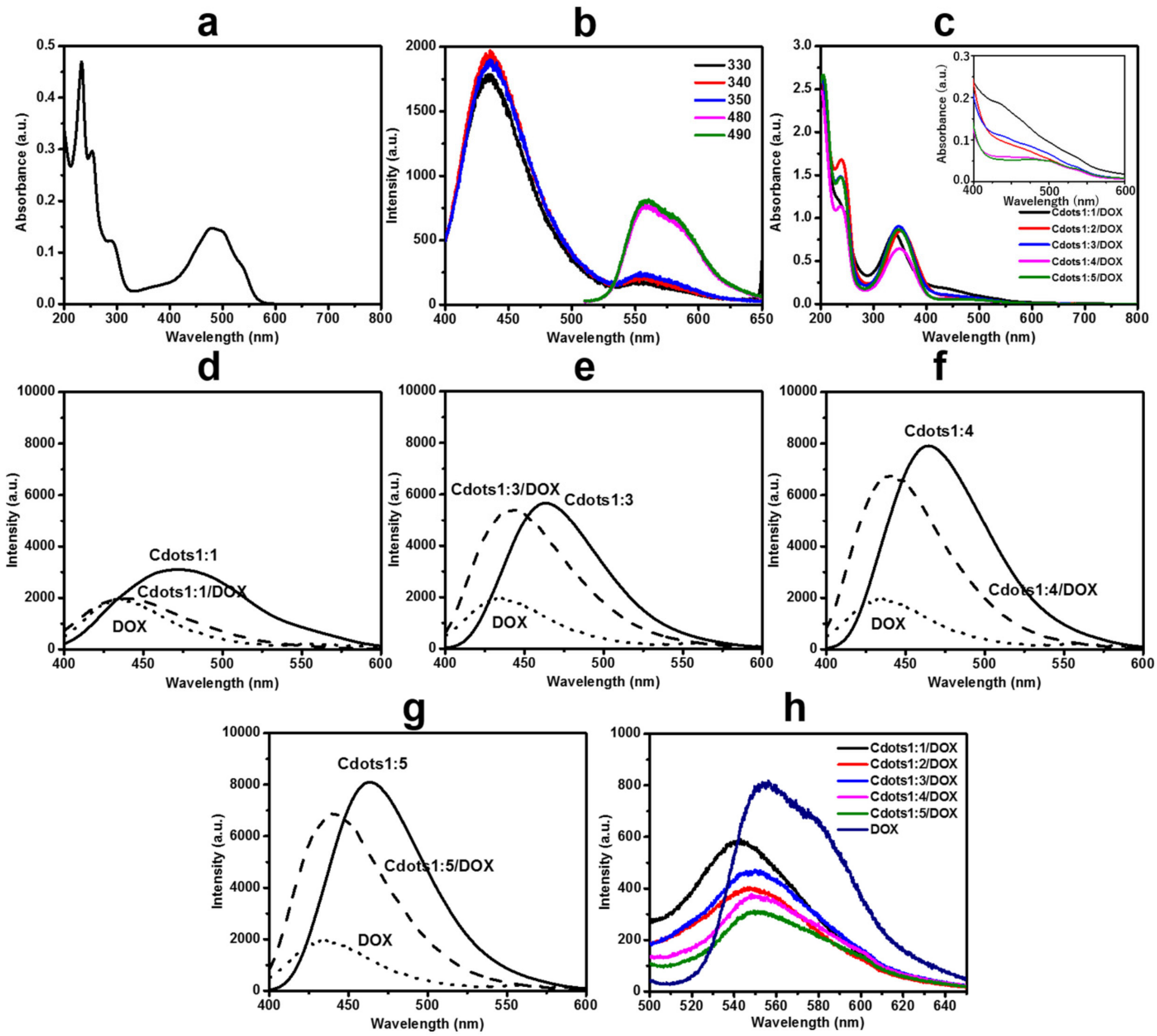

Figure S3. (a) UV-visible absorption spectrum of DOX, (b) emission spectra of DOX at different excitation wavelengths, (c) UV-visible absorption spectra of Cdots1:x/DOX, emission spectra of (d) Cdots1:1(solid line), Cdots1:1/DOX (dashed line) and DOX (dotted line), (e) Cdots1:3, Cdots1:3/DOX and DOX, (f) Cdots1:4, Cdots1:4/DOX and DOX, (g) Cdots1:5, Cdots1:5/DOX and DOX at excitation wavelength 340 nm, and (h) Cdots1:x/DOX and DOX at excitation wavelength 490 nm.

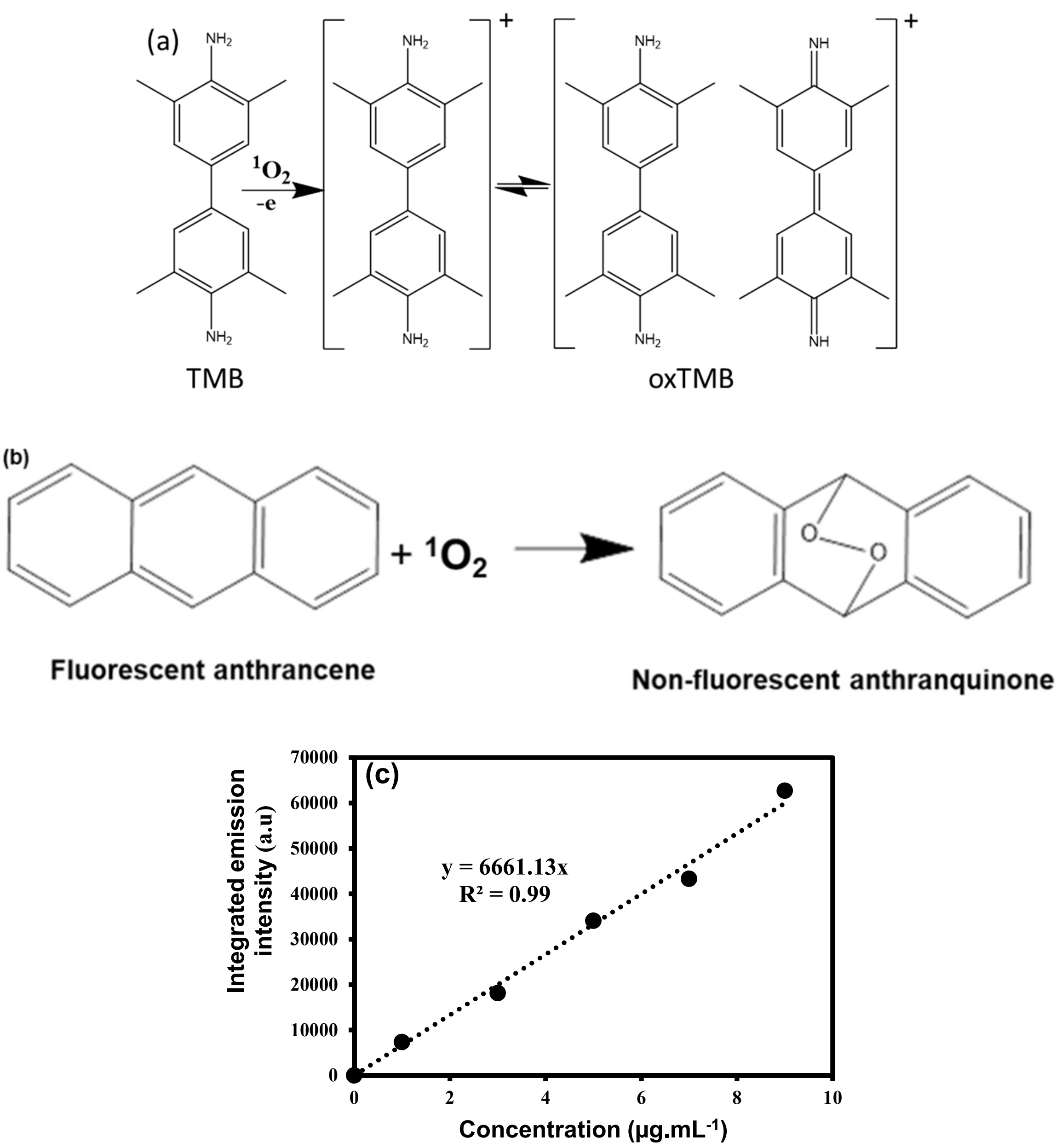

Figure S4. (a) A possible oxidation pathway of TMB by ROS, (b) a reaction of anthracene with singlet oxygen and a plot of mission intensity of antracene as a function of antracene concentration.

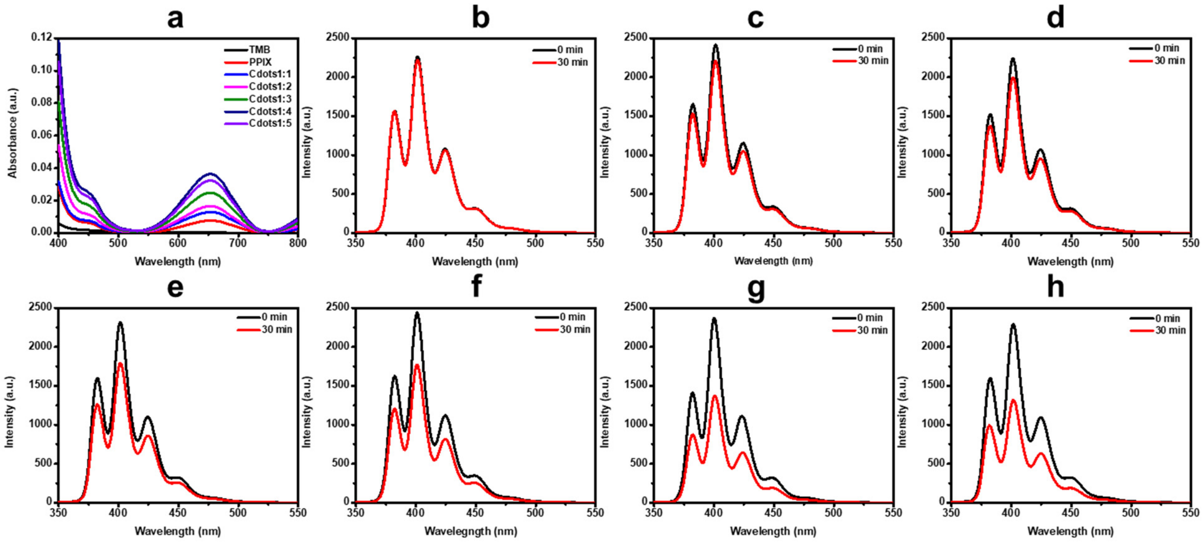

Figure S5. (a) UV-visible absorption spectra of TMB and TMB in the presence of materials after 20 min irradiation, emission spectra of (b) anthracene solution, anthracene solution in the presence of (c) PPIX, (d) Cdots1:1, (e) Cdots1:2, (f) Cdots1:3, (g) Cdots1:4, and (h) Cdots1:5.

Table S2. IC_{50} values of materials.

Sample	IC_{50} ($\mu g\cdot mL^{-1}$)	
DOX	0.078	HeLa cells (This work)
Cdots1:1/DOX	0.765	
Cdots1:2/DOX	0.031	
Cdots1:3/DOX	0.114	
Cdots1:4/DOX	0.104	
Cdots1:5/DOX	0.243	
DOX	0.939	MCF-7 cells[1]
Cdots/DOX	0.356	
DOX	2.94	MGC-803 cells[2]
Cdots/DOX	3.17	
DOX	1.77	MCF-7 cells[3]
N-Cdots/DOX-	0.97	

References

1. Kong, T.; Hao, L.; Wei, Y.; Cai, X.; Zhu, B., Doxorubicin conjugated carbon dots as a drug delivery system for human breast cancer therapy. *Cell Proliferation* **2018,** *51*, e12488.
2. Duan, Q.; Ma, Y.; Che, M.; Zhang, B.; Zhang, Y.; Li, Y.; Zhang, W.; Sang, S., Fluorescent carbon dots as carriers for intracellular doxorubicin delivery and track. *J. Drug Deliv. Sci. Technol.* **2019,** *49*, 527-533.
3. Frieler, M.; Pho, C.; Lee, B. H.; Dobrovolny, H.; Akkaraju, G. R.; Naumov, A. V., Effects of Doxorubicin Delivery by Nitrogen-Doped Graphene Quantum Dots on Cancer Cell Growth: Experimental Study and Mathematical Modeling. *Nanomaterials (Basel)* **2021,** *11* (1), 140.
